# Adaptive Connectivity Restoration from Node Failure(s) in Wireless Sensor Networks

**DOI:** 10.3390/s16101487

**Published:** 2016-09-28

**Authors:** Huaiyuan Wang, Xu Ding, Cheng Huang, Xiaobei Wu

**Affiliations:** Academy of Automation, Nanjing University of Science and Technology, Nanjing 210094, China; why5841031@163.com (H.W.); xdnjust@163.com (X.D.); wuxb@njust.edu.cn (X.W.)

**Keywords:** wireless sensor networks, cooperative communication, connectivity restoration

## Abstract

Recently, there is a growing interest in the applications of wireless sensor networks (WSNs). A set of sensor nodes is deployed in order to collectively survey an area of interest and/or perform specific surveillance tasks in some of the applications, such as battlefield reconnaissance. Due to the harsh deployment environments and limited energy supply, nodes may fail, which impacts the connectivity of the whole network. Since a single node failure (cut-vertex) will destroy the connectivity and divide the network into disjoint blocks, most of the existing studies focus on the problem of single node failure. However, the failure of multiple nodes would be a disaster to the whole network and must be repaired effectively. Only few studies are proposed to handle the problem of multiple cut-vertex failures, which is a special case of multiple node failures. Therefore, this paper proposes a comprehensive solution to address the problems of node failure (single and multiple). Collaborative Single Node Failure Restoration algorithm (CSFR) is presented to solve the problem of single node failure only with cooperative communication, but CSFR-M, which is the extension of CSFR, handles the single node failure problem more effectively with node motion. Moreover, Collaborative Connectivity Restoration Algorithm (CCRA) is proposed on the basis of cooperative communication and node maneuverability to restore network connectivity after multiple nodes fail. CSFR-M and CCRA are reactive methods that initiate the connectivity restoration after detecting the node failure(s). In order to further minimize the energy dissipation, CCRA opts to simplify the recovery process by gridding. Moreover, the distance that an individual node needs to travel during recovery is reduced by choosing the nearest suitable candidates. Finally, extensive simulations validate the performance of CSFR, CSFR-M and CCRA.

## 1. Introduction

Numerous applications of wireless sensor networks has led to much research work recently [1]. For some applications, such as urban search and rescue, space exploration, battlefield surveillance, forest fire detection and containment, it is expected that a set of mobile sensor nodes will be employed to monitor the area of interest collaboratively. The unattended operation of these sensors in the harsh environment, avoids the risks to human life and decreases the cost of the applications. Normally, sensors in these applications would be battery-operated with limited energy, processing and communication capabilities. After deploying the sensors, they are envisioned to form a network through self-organization so that they can communicate with each other and deliver the sensed data to the sink node. To ensure such interactions, nodes need to stay reachable with each other, so the connectivity becomes the bottommost requirement of the network.

Nevertheless, nodes are prone to fail due to the harsh deployed environments and limited energy supply. The loss of node(s) can break the communication paths and divide the network into disjoint blocks, thus the performance of the whole network will be influenced. Therefore, it is necessary to detect the failure(s) and restore the connectivity of the network. Since the WSN usually operates autonomously in the unattended environments, the recovery procedure should be a distributed self-healing process. Moreover, the connectivity restoration should be quick and lightweight. Rapid restoration is desirable in order to maintain network responsiveness to detect events. Given the constrained resources of sensor nodes, the overhead should be minimized. As mentioned before, the greatest challenge is to handle node failures, which may cause the partition of the network. The main issue for restoring connectivity in such cases is that the number of remaining nodes may be too small to restore the connectivity in some local areas.

In conventional non-cooperative networks, nodes can communicate with each other directly within the distance $R_{c}$ (i.e., the communication range), which is determined by the required received signal-noise-ratio (SNR), the peak transmission power, and the path loss attenuation function. One of the methods to ensure network connectivity is using the network redundancy and constructing a k-connected network topology [2,3,4] during the network self-organization phase, such as 2-Connected Relay Node Double Cover problem (2CRNDC) [5]. Moreover, many previous studies utilize the character of node mobility and find a solution of single node failure, especially the failure of a cut-vertex, such as Distributed Actor Recovery Algorithm (DARA) [6], Partition Detection and Recovery Algorithm (PADRA) [7], Volunteer-instigated Connectivity Restoration (VCR) [8], distributed partitioning Detection and Connectivity Restoration algorithm (DCR) [9], distributed algorithm for Recovery through Inward Motion (RIM) [10], Application-oriented Fault Detection and Recovery algorithm (AFDR) [11] and Length-Aware Topology Reconfiguration Algorithm (LTRA) [12]. However, most only focus on the failure of a single cut-vertex and ignore the influence of normal node failure, which is not a cut-vertex failure. Meanwhile, Tian et al. [13] proposed a greedy algorithm to construct a barrier in heterogeneous WSN by adopting weather forecast. Static regular and mobile robust sensors are deployed in the network. The mobile nodes are used to construct another barrier when the weather changes. However, this paper aims to solve the network connectivity restoration problems in all kinds of application with cooperative communication and node mobility.

Certainly, there are several existing approaches, such as MPADRA (which is the expansion of PADRA to handle multiple simultaneous node failures) [7], RAM (which is an extended version of DCR to handle one possible case of a multi-actor failure) [9], distributed algorithm for Autonomous Repair of damaged WSN topologies (AuR) [14], Distributed algorithm for Optimized Relay node placement using Minimum Steiner tree (DORMS) [15] and Shortest Cheapest Path algorithm (SCP) [16], which deal with the failures of multiple node. However, MPADRA and RAM only handle the failures of multiple critical nodes (i.e., cut-vertex). They do not consider the influence of multiple normal node failures. On the other hand, AuR, DORMS and SCP consider nothing but the problem when the network is partitioned due to the multiple node failures. Nevertheless, network partition is only one of the issues that the multiple node failures may lead to. Thus, the algorithms above may not be suitable for all cases of multi-node failure.

Recently, cooperative Multiple Input Multiple Output (MIMO) [17], which is a communication model, has been used to increase channel capacity and reduce transmission energy consumption in wireless ad hoc networks. Because of the restriction of energy efficiency in large wireless sensor networks, the concept of virtual MIMO attracts a growing interest. In virtual MIMO networks (i.e., cooperative networks), nodes can establish connecting paths to nodes outside of their communication range $R_{c}$ with the cooperation of neighboring nodes. The asymptotic connectivity properties have been discussed by Goeckel et al. [18]. Considering the above analysis comprehensively and in order to address the drawbacks of the previous algorithms, this paper presents Collaborative Single Node Failure Restoration algorithm (CSFR), Collaborative Single Node Failure Restoration algorithm with node Mobility (CSFR-M) and Collaborative Connectivity Restoration Algorithm (CCRA). All three of these algorithms are applied in the cooperative networks where a set of mobile sensor nodes is deployed. The main contributions of this paper are:

1. Unlike most previous studies, CSFR restores the network connectivity via cooperative communication after an arbitrary single node fails (not only cut-vertex). Then, CSFR-M, which is the extension of CSFR, is presented to solve the problem of single node failure with the combination of cooperative communication and node mobility. With the combination of these two technologies, CSFR-M can overcome the shortcomings of CSFR and address the drawbacks of the existing mechanisms.
2. CCRA is proposed to handle all of the problems of multiple node failure, not only the problems of multiple cut-vertex failure and network partition caused by the failures. CCRA is a localized and reactive scheme that limits the scope of node movements and the energy consumption of each node during the recovery process.
3. In order to finish the recovery process in small scopes, CCRA divides the network into grids during the topology self-organization. The main idea is to reestablish the disconnected paths that are caused by the node failures via cooperative communication and the movement of some suitable sensor nodes. The principle is that the involved nodes only move out of corresponding grid when certain scenarios occur.
4. The main properties of CSFR-M and CCRA are their simplicity and effectiveness. CSFR-M and CCRA avoid sophisticated diagnostics for evaluating the effects of node failure on the network connectivity, e.g., by determining whether the failed node is a cut-vertex or not. The entire process is distributed and enables the network to be self-healing without any external supervision.

The rest of the paper is organized as follows. The next section reviews the related work. In Section 3, we describe the considered system model, highlight the implications of node failures on the network topology and give the formulation of the problem. Section 4 provides the detailed description and analysis of the proposed CSFR-M and CCRA algorithms. Section 5 gives the analysis of the CSFR-M and CCRA algorithms. Validation simulations and performance analysis are provided in Section 6. Finally, we give a conclusion of this paper in Section 7.

## 2. Related Work

In this section, we will review the related work briefly. Given the importance of connectivity in wireless sensor networks (WSNs), many researchers [2,19] are focused on connectivity restoration from network disconnections caused by single node failure. Generally, the existing schemes can be classified into two categories according to the functional principle: proactive and reactive.

Proactive methods require redundant resources to make up for the loss of a failed node. K-connectivity [3,4,20] is proposed to solve this problem. There are K-disjoint paths between any pair of nodes; hence, the algorithms can tolerate the failure of K-1 members. In order to achieve a better fault-tolerant property, 2CRNDC [5] strives to maintain a two-connected topology network with minimized number of relay nodes. Even when the number of relay nodes is restrained, it still requires no connectivity restoration because of its redundancy. Deniz et al. [21] proposed an adaptive, energy-aware and distributed fault-tolerant topology-control algorithm, namely the Adaptive Disjoint Path Vector (ADPV) algorithm for heterogeneous WSNs. There are two kinds of nodes in this heterogeneous network: resource-rich super-nodes and ordinary nodes. ADPV strives to construct and maintain a k-vertex super-node connectivity topology to prolong the lifetime of k-vertex super-node connected network. In addition, ADPV is an adaptive approach, adapting to node failures and remaining energy levels. The focus of ADPV is to ensure super-node connectivity in the presence of node failures, and ADPV achieves this goal by dynamically adjusting the sensor nodes’ transmission powers. Similar to all the K-connectivity solutions, it requires the redundancy of the network and can only tolerate K-1 node failures. Moreover, the ADPV demand that all super-nodes communicate with all sensors directly is hard to achieve.

Different from the proactive methods, the recovery processes are initiated once the failure is detected in reactive solutions. The basic idea is to replace the failed node with suitable backup node through inward movement when the network is partitioned, and it has been deemed as an efficient strategy in most of the recent studies. For instance, DARA [6] pursues a coordinated relocation to restore the broken communication paths among the neighbors of the failed node. With the information of two-hop neighbors, DARA can identify whether a node is a cut-vertex or not. Once a failure happens, DARA selects the best candidate (BS) from the neighbors of the failed node according to their degree, distance, and node ID, and then moves the BS to the failed node’s position to restore the connectivity. If any neighbor of BS cannot communicate with the BS after its movement, the neighbors of the BS initiate the process so that a cascaded movement is proposed to solve the problem. Moreover, Akkaya et al. [7] proposed an algorithm named PDARA, which forms a connected dominating set (CDS). PDARA informs a particular node in advance whether a partition will occur or not in the case that it fails (i.e., cut-vertex identification). Once a cut-vertex fails, a failure handler (FH) of the cut-vertex will initiate the recovery process. The FH finds the closest node that is dominated by the failed node and uses it as a replacement of the failed node. The overall goal is to localize the scope of the restoration and minimize the distance of movement. In order to minimize the message overhead, DCR [9] identifies the critical nodes with one-hop information and designates backups for them. The replacement is similar to DARA.

As mentioned, all these three algorithms are focused on the single node (cut-vertex only) failure problem and relocate the designated backup to the position of failed node. Unlike DARA, etc., another notable work on connectivity restoration named RIM is proposed by Younis et al. [10]. All the 1-hop neighbors move towards the position of the failed node till the distance is “$R_{c}/2$”, which $R_{c}$ is the uniform communication range of all the sensor nodes. Other nodes carry out a cascaded inward movement if they cannot communicate with the moved nodes. Although the distance that the individual node has to travel is small, the number of involved nodes in RIM may be huge in the case the area is deployed with large number of nodes. Recently, a novel method named LTRA is proposed by Zhang et al. [12]. LTRA is focused not only on critical nodes but also on the nodes whose failure may increase the number of the critical nodes. However, they all deal with the problem of single node failure.

Certainly, there are some studies focused on the problems of multi-node failure. Akkaya et al. [7] proposed MDAPRA, which is a modified algorithm of PDARA, and it strives to reposition the nodes to recover from multiple cut-vertex failures via some mutual exclusion mechanism. In the case two or more cut-vertices fail around the same time, each FH of the failed cut-vertex will initiate the recovery process and they may compete to use the same nodes for repositioning. Every cut-vertex has two failure handlers, primary FH(PFH) and second FH(SFH), in MDAPRA. When PFH cannot find a suitable candidate that has not been reserved and get stuck, the SFH will take over the recovery process after a certain time period $\tau (\tau >\left(\frac{r}{s}+2\left(p+t\right)\left(n-2\right)\right))$. Like MDAPRA, the RAM [9] algorithm, which is based on DCR, imposes additional constraints while choosing backups for critical nodes and avoids to create another network partition during the recovery. The failures of multiple cut-vertex are one of the many situations contained in the problems of multiple node failure, so the algorithms are not suitable for all cases of the multiple node failure.

Moreover, Joshi [14] and Lee [15] focused on the recovery from network partition after multiple node failures. Joshi et al. [14] proposed an autonomous repair algorithm (AuR) to restore network connectivity. AuR is based on the principle that the connectivity between neighboring nodes is modeled as a modified electrostatic interaction based on Coulomb’s law. Self-spreading is proposed based on the rationale of electrostatic attraction and repulsion in AuR. The connectivity is re-established through self-spreading and motion towards the center of the area. DORMS [15] strives to re-connect the blocks after failures of multiple node with a subset of surviving relay nodes in each block. The relays are populated in the shortest path from every block towards the center. Then, the paths are simplified by utilizing the principle of Steiner Minimum Tree (SMT), so that the number of involved relays in the recovery could be minimal. Since the multiple node failures may not always cause a network partition and these two algorithms solve only one situation of the multi-node failures, they may be not suitable to handle all cases.

Similar to DORMS, Truong et al. [16] solve the connectivity restoration problem with the routing planning algorithms. However, SCP [16] is proposed in consideration of obstacles on the accessibility paths of the potential locations. With the knowledge of the connectivity graph $G_{C}$ and the mobility graph $G_{M}$, SCP finds the minimal number of required relays to connect all the terminals based on the Steiner-SMT algorithm, and then it finds the cheapest circuit for the agent to visit all those nodes (including terminals and Steiner nodes). For comparison, Truong et al. present the integrated path algorithm (IP). The IP approach attempts to unite the two objectives, the number of nodes required and the mobility cost, to achieve a better performance in some scenarios. SCP and IP aim to gather information from all of the terminals regularly by a mobile agent with the designed path circuit. Thus, the period of the gathering could be too long for the network and affect the real-time performance.

## 3. Network Model and Problem Formulation

### 3.1. Network Model

Traditionally, sensor nodes in WSN communicate with each other using the disk model, i.e., the sensing region and communication region of the sensors are the circles centered on the sensors with the sensing radius $R_{s}$ and communication radius $R_{c}$, respectively. Sensor nodes are equipped with a single antenna and they can communicate with each other only if the Euclidean distance between any two of them is less than the communication radius, i.e., $d_{ij}\leq R_{c}$ where i and j represent the two nodes.

Nevertheless, the Cooperative Communication technology that has been proposed recently allows single antenna devices to take advantage of the benefits of MIMO system [22]. Generally, the link from node A to node B is available if and only if the received average signal-to-noise ratio (SNR) of node B from node A is not less than a fixed threshold τ, i.e., SNR≥τ, and vice versa. Node A and node B are connected if and only if the link from A to B and the link from B to A are both available. Considering this assumption, we utilize the cooperative communication model refers to [18]. Node i can communicate with node j if and only if the received average SNR of node j from node i ($\beta_{ij}$) is not less than τ, i.e.,

(1) $$\frac{P_{i}E\left[{\left|h_{ij}\right|}^{2}\right]}{{\left(d_{ij}\right)}^{\partial }N}=\;\beta_{ij}\;\geq \;\tau ,\;P_{i}\leq \;P_{0},$$

Assume that node i works with the Power $P_{i}$ to communicate with node j and $P_{0}$ is the maximum transmission power of all the nodes. $h_{ij}$, which is generated by a Rayleigh distribution, is the channel coefficient from node i to node j and $E\left[{\left|h_{ij}\right|}^{2}\right]$ is the channel gain; the distance between node i and node j is denoted by $d_{ij}$ and ∂ is the path loss exponent; and N is the noise power. In this case, we consider node i as the source node and node j as the destination node, and vice versa.

With the cooperative communication technology, when multiple nodes send the same package to the same destination simultaneously, the destination node (node j) will receive multiple signals at the same time. To decode the signals, the well-known method maximal-ratio combining MRC, which is designed for diversity combining in wireless communication and CC studies [23], is utilized. The total SNR that node j received in the output of MRC combiner can be described as the sum of received average SNRs:

(2) $$\beta_{j}=\;\sum_{i\in \varphi }\beta_{ij},$$

φ denotes all the nodes which send the same package. Therefore, the total SNR that node j received must satisfy:

(3) $$\beta_{j}=\;\sum_{i\in \varphi }\beta_{ij}=\;\sum_{i\in \varphi }\frac{P_{i}E\left[{\left|h_{ij}\right|}^{2}\right]}{{\left(d_{ij}\right)}^{\partial }N}\;\geq \;\tau ,\;P_{i}\;\leq \;P_{0},$$

In cooperative communication model, node i and node j are considered connected when the received SNR $\beta_{j}$ of node j is not less than τ and vice versa. In some cases, node i may receive insufficient SNR from node j, so that node j cannot establish the cooperative communication link to node i. Then the communication path between node i and j is unidirectional which is irrespective in this paper. It is assumed that node i and j can communicate with each other directly since the cooperative communication link is established.

Assume all the sensors are deployed randomly in the designated area with the uniform transmission power $P_{0}$, a two-dimensional graph G=(V,E) is formed by self-organization. $V=\left(V_{1},V_{2},\ldots ,V_{n}\right)$ is the set of each sensor node and set E indicates all the connected paths between every two nodes which can communicate directly. V(G) and E(G) are the vertex-set and edge-set of graph G, respectively. The network is constructed base on the disk model initially. The set of nodes that node i can communicate with directly is denoted as N(i). In other words, set N(i) represents all the 1-hop neighbors of node i.

**Definition 1** (**Helper Nodes Set**)**.** H(s) *indicates the set of all the helper nodes that help the source node* s *to establish the link with the destination node* d *via cooperative communication*.

**Definition 2** (**Help Links**)**.** *Help links are the direct links between the source node* s *and the nodes in* H(s)*.*

**Definition 3** (**Cooperative Communication Power**)**.** $P_{i}^{c}$ *symbolizes the required cooperative communication power assigned for the source node* s *and all the nodes in* H(s) *when the cooperative communication path between source node* s *and destination node* d *is established, where* i∈φ *and* φ *is the union set of source node* s *and* H(s)*.*

**Definition 4** (**Node Connected Degree**)**.** $D_{i}$ *indicates the number of 2-hop neighbors of node* i*.*

**Definition 5** (**Node Connectivity Materiality**)**.** *Node connectivity materiality of node* i *(*$M_{i}$*) is the ratio of the shortest path hops sum between nodes in* N(i) *before and after node* i *fails, i.e.,* Mi= LiL0= ∑j,k∈ψ;j≠kHnjk*∑j,k∈ψ;j≠kHnjk*,* $L_{i}$ *indicates the sum of the shortest path hops when* i *is failed and* $L_{0}$ *is the sum of the shortest path hops in the initial graph,* $Hn_{jk}$ *and* $Hn_{jk}^{*}$ *indicate the shortest path hops from* j *to* k *before and after node* i *fails,* ψ *is the set of neighbor nodes of node* i*, let* $Hn_{jk}^{*}=\infty$ *when two nodes cannot communicate with each other due to the failure.*

**Definition 6** (**Node Partition Character**)**.** *Node partition character of node* i $\left(PC_{i}\right)$ *is the number of disconnected blocks after the failure of node* i*. Let the node partition character of node* i*,* $PC_{i}=0$*, when the node connectivity materiality* $M_{i}\neq \infty$ *; otherwise,* $PC_{i}\geq 2$*.*

Assume every node knows its location and each node has a unique ID. All the nodes in WSN exchange their location information and node ID during the self-organization phase. Since the network topology is formed, each node keeps a neighbor table, which contains the location information ID of all the 2-hop neighbor nodes, the connectivity materiality and partition character.

### 3.2. Problem Formulation

Generally, sensors in WSN may be deployed in the harsh environment and form the network by self-organization. Sensor nodes are prone to fail in hostile environment because of battery exhausting, hardware faults, etc. The network connectivity, which is the bottommost requirement to guarantee the availability of the network, will be destroyed due to the node failure(s). Most of the previous studies are devoted to solving the problem of single cut-vertex failure, whereas this paper discusses the problems of single-node (whether it is a cut-vertex or not) and multi-node failures.

**Figure 1:**
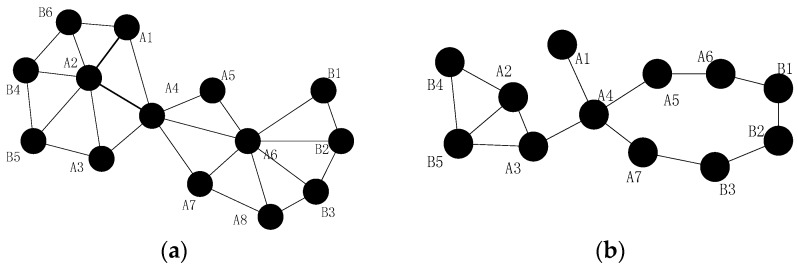
(**a**) Cut-vertex failure; and (**b**) normal node failure.

As discussed previously, the failure of a cut-vertex will destroy the network connectivity since it divides the network into disconnected regions, as shown in Figure 1a. If node A4 fails for some reason, the network will be divided into two blocks and nodes in different blocks cannot communicate with each other. In this case, the network is doomed since the network connectivity is destroyed. Because node A1 and A2 are out of the communication range of node A5 and A6, they cannot establish communication path with each other in normal communication model (i.e., disk model). However, they can re-establish the communication path via cooperative communication. For instance, node A2 may use its neighbors (node A1, A3, B4 and B5) as helper nodes to build the connecting path to node A6. In addition, node A6 will recover the connectivity between node A2 in the same way as mentioned above.

Nevertheless, the failed node may not be a cut-vertex (Case 2 for short), as shown in Figure 1a,b. The failure of node A6 will not affect the network connectivity, but the path length of node A5 and B1 is significantly longer than it was before the failure (from 2 hops to 6 hops), which may cause unnecessary energy consumption. Moreover, many other nodes become cut-vertices due to the failure of node A6, such as nodes A7, A8, etc. As we all know, the load of the paths that contain cut-vertices may be extremely high. The cut-vertices in these paths may consume much more energy than other nodes, and the whole network may be disconnected in a short time. In addition, the traffic interference of the paths will be catastrophic, the communications may be difficult and the network will not be able to work successfully. Similarly, when node A2 fails in Figure 1a, all of its neighbors can still communicate with each other, but the shortest path has changed. Thus, it is also important to solve this problem and not only the cut-vertex failure.

In consideration of these two cases, we can formulate the problem of single node failure restoration via cooperative communication as follows:

**Problem** **1.** *Given a self-organized and connected network* G=(V,E)*, when a single node* i *fails, assign a power level to the involved nodes so that: (1) the network connectivity is restored; (2) the node connected degree of* N(i) *is non-decreasing; and (3) the sum of the assigned cooperative communication power is minimum*.

The topology control with cooperative communication problem (TCC) is proven NP-complete in [23]. The above Problem 1 is a particular case of TCC, which only requires the involved neighboring nodes to be CC-based connected. Thus, it is also NP-complete, and it is necessary to have an energy-efficient algorithm that maintains the network availability via cooperative communication to solve the problem. Let $P_{s}^{d}\left(i\right)$ be the minimum required power for source node s to communicate with node i directly, where i ∈H(s) and the superscript d stands for the direct communication. It is also the minimum required power for the help link (s,i). Symbol $P_{s}^{c}\left(d\right)$ and $P_{i}^{c}\left(d\right)$ are the required cooperative communication power assigned for source node s and helper node i to establish cooperative communication link with destination node d, respectively, and the superscript c stands for the cooperative communication. Since the number of helper nodes may change, the assigned power $P_{s}^{c}\left(d\right)$ and $P_{i}^{c}\left(d\right)$ will be different. Moreover, the failed node may have some neighbors to be selected as source and destination node, different combination will cause distinct power assignment. Taking this tradeoff into consideration, some energy-efficient strategies, which select the appropriate source node, helper nodes and destination node to minimize the sum of assigned transmission power, should be proposed.

Given a connected graph, G=(V,E), V(G) ≠ ϕ and E(G) ≠ ϕ. Each node is deployed with the uniform initial power $P_{0}$. If a node fails, some nodes will be selected as source node s and destination node d. Symbol $P_{s}^{d}\left(i\right)$ is the required communication power for node s to communicate with node i(i ∈H(s)) directly. Without loss of generality, we assume that the channel gain $E\left[{\left|h_{ij}\right|}^{2}\right]=1$ and the noise power N=1 in the previous formulas to reduce complex notation. Therefore, according to Equations (1) and (3), we can obtain the following:

(4) $$P_{s\;\cup H\left(s\right)}^{c}\left(d\right)=\;P_{\varphi }^{c}\left(d\right)=\;\frac{\tau }{\sum_{i\;\in \;\varphi }{\left(d_{id}\right)}^{-\partial }},$$

(5) $$\underset{i\;\in H\left(s\right)}{\max}P_{s}^{d}\left(i\right)=\;\frac{\tau }{{\left(\underset{i\;\in H\left(s\right)}{\max}d_{si}\right)}^{-\partial }},$$

Symbol $P_{s\;\cup H\left(s\right)}^{c}\left(d\right)$ is the minimum assigned cooperative power for source node s and every helper node i ∈H(s) to establish cooperative communication link with destination node d collectively. Symbol $\underset{i\;\in H\left(s\right)}{\max}P_{s}^{d}\left(i\right)$ is the minimum assigned power for source node s to communicate with every helper node i ∈H(s) directly. Thus, problem 1 can be formulated as follows:

(6) $$Minimize\;\sum_{j\in \varphi }P_{j}\left(d\right)=\;P_{s}\left(d\right)+\;\sum_{i\;\in H\left(s\right)}P_{i}\left(d\right),$$

$$\begin{array}{ll} s.t.\;\varphi =s\;\cup H & \left(s\right);\\ & \sum_{j\;\in \;\varphi }\frac{P_{j}}{{\left(d_{jd}\right)}^{\partial }}\;\geq \;\tau ;\\ & P_{s}\left(d\right)=\max\left\{\underset{i\in H\left(s\right)}{\max}P_{s}^{d}\left(i\right),\;P_{\varphi }^{c}\left(d\right)\right\};\\ & P_{i}\left(d\right)=\max\left\{P_{i}^{d}\left(s\right),\;P_{\varphi }^{c}\left(d\right)\right\};\\ & \sum_{n\;\in N\left(f\right)}D_{n}\;\leq \;\sum_{n\;\in N\left(f\right)}(D_{n}^{*}+1). \end{array}$$

The first constraint denotes that set φ is the union set of source node s and the helper nodes set H(s). Assume that the source node s and the helper nodes in H(s) transmit the same package to the destination node d with the same cooperative power simultaneously. The second constraint ensures that there are enough helper nodes to establish a cooperative communication link (denotes as CC-link for short hereafter) between source node and destination node as shown in Equation (3). In order to establish the CC-link, node s will have two power levels when building the CC-link: the power for communicating with the helper nodes directly and the assigned cooperative power. Similarly, the helper nodes will have the power for communicating with the source node directly and the assigned cooperative power. Then, the third and fourth constraints guarantee that the source node and the helper nodes are assigned with a minimum suitable power to maintain the CC-link. As mentioned above, the Case 2 of node failure is also considered in this paper and the fifth constraint ensures that. When a node f fails for some reason, $D_{n}^{*}$ is the connected degree of its neighbor node n (n ∈N(f)) after the cooperative communication path is established and $D_{n}$ indicates the connected degree before the failure. Since the failed node is removed from the network, the connected degree of its neighbors will decrease by at least one, regardless of whether the link is reestablished. In Case 2 of single node failure, the connected degree of its neighbors will also decrease significantly and the above constraint makes sure that the connectivity is restored.

## 4. Node Failure Restoration via Cooperative Communication

In this section, we present three algorithms: Collaborative Single Node Failure Restoration (CSFR), Collaborative Single Node Failure Restoration with node Mobility (CSFR-M) and Collaborative Connectivity Restoration Algorithm (CCRA) in detail.

### 4.1. Collaborative Single Node Failure Restoration (CSFR)

Similar to our proposed algorithm CCFR [24], CSFR is a distributed algorithm in which the involved nodes restore the connectivity cooperatively. As described previously, the neighbors of the failed node will initialize the recovery process after detecting the failure. Since the neighbors of the failed node may not be able to communicate with each other when the failed node is a cut-vertex, it is important that all nodes should maintain a 2-hop neighbors table. The 2-hop neighbors table may contain all the 1-hop and 2-hop neighbors, along with the coordinates of all the 1-hop and 2-hop neighbors. Each node will also tabulate the node ID, node connectivity materiality and partition character of all the nodes in the 2-hop neighbors table. CSFR ensures that the restoration procedure is convergent. The following sections describe the detailed steps.

#### 4.1.1. Source and Destination Nodes Selection

Heartbeat messages will be sent periodically by every sensor node to their neighbors to declare that they are functional and also to report changes to the 1-hop neighbors. Thus, the failure of a node will be detected if all of its neighbor nodes miss the heartbeat message from it. Depending on the node connectivity materiality, the neighbors of the failed node may decide whether a recovery is needed or not. As discussed previously, the failure of a node that does not impede the connectivity of any other nodes would not necessitate any restorations to the network topology. When a node f fails, the neighbors of node f will check the node connectivity materiality of f: (1) if $M_{f}=1$, no recovery is needed; (2) if $1<M_{f}<\infty$, the failed node f is not a cut-vertex, but the shortest path between its neighbors has increased, i.e., the Case 2 discussed above; and (3) if $M_{f}=\;\infty$, it is obvious that the network connectivity has been destroyed.

**Figure 2:**
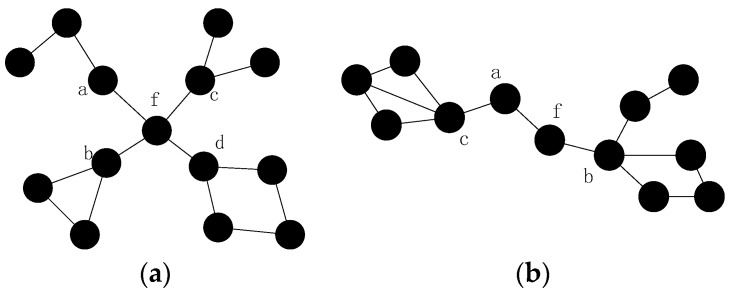
(**a**) Multiple disconnected blocks; and (**b**) linear node failure.

As shown in Figure 1a,b, when node A6 fails, the shortest path between its neighbors increased, so it is necessary to decrease the increment. Since the shortest path between node A5 and B1 is the longest among all the others after failure, CSFR may choose node A5 and B1 as a pair of source and destination nodes when establishing the CC-link. Figure 2a,b shows two cases of single cut-vertex failure, when the network is divided into disconnected regions after node f fails. In Figure 2a, there are four blocks after the failure. Node a, b, c and d will select themselves as the pair of source and destination nodes based on the information of 2-hop neighbors. According to Equations (4) and (5), the two nearest nodes are chosen as source and destination nodes, for example, node a and b. Thus, node a and b will establish connecting path via cooperative communication firstly, the block contains node a and the block contains node b will be referred to as a group after the collaboration. Then, there are three disconnected blocks and the recovery process continues, the second nearest nodes in the different blocks will be selected as source and destination nodes, and the process goes on until the network is connected.

Considering all the analysis above, the 1-hop neighbors of the failed node will initiate the recovery process. The source and destination nodes are selected between them based on the following factors: the distance $d_{sd}$ between each other, the node connected degree and the node ID. The two nodes with a shortest distance $d_{sd}$, which is greater than the communication range $R_{c}$, will be selected as a pair of source and destination nodes. When the shortest $d_{sd}$ is equal, the nodes with the biggest node connected degree will be preferred. The pair of nodes with the smallest node ID will choose themselves as the source and destination nodes when the above two factors cannot be judged. Of the source and destination node pair, whichever is closer to the failed node f will choose itself as the source node and start following helper nodes selection first. The other node, i.e., destination node, will start a timer ($T_{b}$) to wait for the data packet from the source node. The roles will be exchanged between them so as to build the bidirectional CC-link as discussed before.

#### 4.1.2. Helper Nodes Selection

Again, since the CC-link is bidirectional, the source and destination nodes will select their helper nodes, separately. The helper nodes selection will be explained using the source node as an example. According to Equations (4) and (5), the cooperative communication power $P_{s\;\cup H\left(s\right)}^{c}\left(d\right)$ mainly depends on the distance between the nodes in s∪H(s) and the destination node. Let $D_{H}^{d}$ denote the distance between the direct neighbors of source node (i.e.,N(s)) and destination node. Then, the source node adds neighbor nodes into the helper nodes set H(s) in ascending order of the distance $D_{H}^{d}$. The source node will check whether Equation (3), which is the primary principle for a CC-link, is satisfied or not once a helper node is added. Upon computation, the source node will set the appropriate nodes into the helper nodes set.

#### 4.1.3. Cooperative Communication

For the source node, it will decide whether the CC-link establish requirement has been satisfied. If not, the source node will give up the establishment and inform all its neighbors, which may contain some of the 1-hop neighbors of the failed node f, about the failure of CC-link buildup. Otherwise, having selected the helper set and assigned corresponding communication powers, it will start cooperative transmission by sending the data packet to all the helper nodes. Then all the nodes in the cooperation set cooperatively transmit the data packet to the destination node over orthogonal channels, such as using Code Division Multiple Access (CDMA) or Time Division Multiple Access (TDMA), at the assigned powers [25]. Once the data packet is sent, the source node will start the same timer ($T_{b}$) and act as a destination node.

As for the destination node, it will start the timer ($T_{b}$) to wait for the data packet from the source node first. If it receives the data packet before the timer is expired, the destination node will start the helper nodes selection and act as a source node. If the timer expires and the destination node receives nothing, the destination node will inform all of its neighbors, which may contain some of the 1-hop neighbors of the failed node f, about the failure of CC-link buildup. The timer ($T_{b}$) may contain the computation time $T_{c}$ for helper nodes selection and the data packet transmission time $T_{p}$, i.e., $T_{b}=T_{c}+T_{p}$ [26].

In the worst and rare case, such as node f in Figure 2a, suppose the CC-link is established following the order of a→b→d→c. Since all of the neighbors of node f know the $PC_{f}$, the node d and c will set a timer $\left(PC_{f}-1\right)\times 2T_{b}$ and wait for CC-link establishment. After node a and b have finished the establishment, they will try to build the CC-link with node d, and the algorithm continues until the network is connected again. If some CC-links cannot be built, such as the CC-link between node a and b, they will back off and announce the failure of the CC-link establishment. The other nodes will find out the infeasible establishment when the timer expires. The pseudo-code of CSFR is shown as Algorithm 1:
**Algorithm 1** CSFR**1**: f: the failed node; $N_{f}$: the 1-hop neighbors of node f**2**: $M_{f}$: the node connectivity materiality**3**: **if**
$1<M_{f}<\infty$
**then****4**: **for** every node j and l in $N_{f}$, which j≠l
**do****5**:  **if** the distance $d_{jl}>R_{c}$ and $d_{jl}$ is the shortest distance among the 1-hop neighbors **then****6**:   u←the source node, d←the destination node;**7**:  **end if****8**: **end for****9**: Algorithm 2 CC-link establishment**10**: **end if**
**Algorithm 2** CC-link establishment**1**:  f: the failed node; $N_{f}$: the 1-hop neighbors of node f**2**:  $M_{f}$: the node connectivity materiality**3**:  u: the source node; d: the destination node**4**:  $A=\left\{N_{1},N_{2},\ldots ,N_{m}\right\}$ includes all the neighbor nodes of u; m: the total number of neighbors of u**5**:  $H_{\left(u\right)}$: the helper set of source node u; φ: the cooperation set**6**:  $H_{\left(u\right)}\leftarrow \phi ,\;\varphi \leftarrow u,\;k\leftarrow 0$**7**:  **while**
$\sum_{i\in \varphi }P_{i}^{c}{\left(d_{id}\right)}^{-\partial }<\tau$ and k<m
**do****8**:   $k\leftarrow k+1;\;H_{\left(u\right)}\leftarrow H_{\left(u\right)}\cup N_{k};\;\varphi \leftarrow \varphi \cup H_{\left(u\right)}$**9**:  **end while****10**: **if**
k=m
**then****11**:   **if**
$\sum_{i\in \varphi }P_{i}^{c}{\left(d_{id}\right)}^{-\partial }\geq \tau$
**then****12**:    Return $H_{\left(u\right)}$, $\sum_{i\in \varphi }P_{i}\left(d\right)$**13**:   **else****14**:     Announce the failure of CC-link establishment**15**:   **end if****16**: **else****17**:   **if**
$\sum_{i\in \varphi }P_{i}\left(d\right)<\sum_{i\in \varphi \cup N_{k+1}}P_{i}\left(d\right)$
**then****18**:    Return $H_{\left(u\right)}$, $\sum_{i\in \varphi }P_{i}\left(d\right)$**19**:   **else****20**:    $k\leftarrow k+1;\;H_{\left(u\right)}\leftarrow H_{\left(u\right)}\cup N_{k};\;\varphi \leftarrow \varphi \cup H_{\left(u\right)}$**21**:   **end if****22**: **end if****23**: the source and destination nodes will interchange and establish the CC-link again in reverse

### 4.2. Collaborative Single Node Failure Restoration with Node Mobility (CSFR-M)

**Figure 3:**
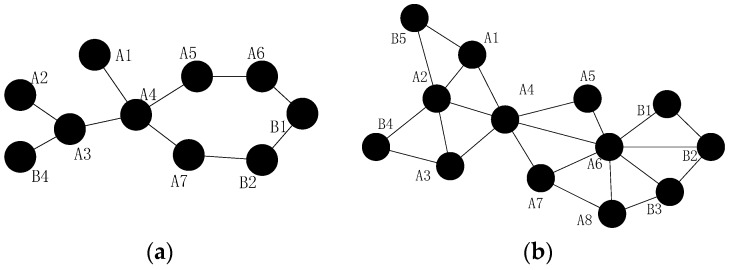
(**a**) With orphan adjacent node; and (**b**) without orphan neighbor.

As mentioned in Section 4.1, the source and destination nodes may not be able to build the CC-link; moreover, the recovery may be complicated in the worst case. To overcome the shortcomings of CSFR, optimize this solution and extend to solve the multiple node failure problems, we will utilize the node mobility as assumed above and propose the optimized algorithm CSFR-M. Assume all of the sensors can move without any movement constraints and the moving energy model of each node is the same (such as the model in [13,27]). As shown in Figure 3a: (1) When node A6 fails, the node A5 and node B1 will be chosen as source node and destination nodes in CSFR respectively. However, node A5 cannot build the cooperative communication link with B1 due to the distance. Thus, in the optimized algorithm CSFR-M, if node A5 fails to build the CC-link, the backup solution will be initialized. Node A5 and B1 will announce the infeasible CC-link establishment and choose a suitable candidate from themselves to replace the failed node. If the neighbors of A5 or B1 lose their communication path with them, they should move towards the new location of A5 or B1 till they can communicate again. The process will keep going until no more links are broken due to the movement; (2) Similarly, node A3 and A7 will be chosen as source and destination node, respectively, when node A4 fails in CSFR. However, the node A7 may not have enough neighbor nodes to establish the CC-link with node A3. Notice that node A1, which is the neighbor node of node A4, is an orphan node and it will move to the location of node A4 to repair the connecting path.

In summary, when the failure of a single node, such as A4 in Figure 3a,b, has been detected, the 1-hop neighbors of the failed node will find out whether there are orphan nodes after the failure. If there is an orphan node, such as node A1 in Figure 3a, A1 will choose itself as the candidate and move to the location of failed node A4; if not, in order to handle the worst case, such as the case in Figure 2a, the 1-hop neighbors of the failed node f will choose a candidate to replace node f directly when the partition character $M_{f}=\infty ,PC_{f}>2$. Otherwise, the 1-hop neighbors of the failed node will choose the corresponding source and destination nodes (A3 and A7) from themselves. As discussed before, the source node (A7 in Figure 3b) will try to build the unidirectional cooperative communication path to node A3 first after selecting respective helper nodes. The destination node (A3 in Figure 3b) will wait for a fixed time $T_{b}$ to receive the data package from node A7. If they cannot restore the connecting path, the process will back off and choose the optimum node(s) to repair the communication.

There are several other scenarios: (1) if the node connectivity materiality of the failed node $M_{f}\;=\;\infty$ and the node partition character $PC_{f}=2$, the node which has the minimum number of 1-hop neighbors (A5 in Figure 3b in this case) will move to the location of the failed node. The neighbors of node A5 will move to keep connecting with it in case the communication path between them is broken. (2) If the node connectivity materiality of the failed node $1<M_{f}<\;\infty$ and there are also no orphans around (as discussed above the failure of node A6 in Figure 3a), then the nodes (A5 and B1) which have been chosen previously will announce the failure of CC-link establishment, and the 1-hop neighbors of node f will choose the node with the minimum number of 1-hop neighbors to replace the failed node. The neighbors of the relocated node will move to keep in touch if necessary. The pseudo-code of CSFR-M is shown as Algorithm 3:
**Algorithm 3** CSFR-M**1**:   f: the failed node; $M_{f}$: the node connectivity materiality; $PC_{f}$: the node partition character**2**:   **if**
$1<M_{f}\leq \infty$
**then****3**:    **if** there are orphan nodes after the failure **then****4**:     the orphan which is nearest to node f will be chosen and move to replace it**5**:    **else****6**:     **if**
$1<M_{f}<\infty$
**then****7**:      Algorithm 2 CC-link establishment**8**:      **if** receiving the announcement **then****9**:       the neighbors $N_{f}$ with minimum number of neighbors will be chosen to replace f**10**:      **end if****11**:     **else****12**:      **if**
$PC_{f}>2$
**Then****13**:       the neighbors $N_{f}$ with minimum number of neighbors will be chosen to replace f**14**:      **else****15**:       Algorithm 2 CC-link establishment**16**:       **if** receiving the announcement **then****17**:        the neighbors $N_{f}$ with minimum number of neighbors will be chosen to replace f**18**:       **end if****19**:      **end if****20**:     **end if****21**:    **end if****22**: **end if**

### 4.3. Collaborative Connectivity Restoration Algorithm (CCRA)

Since the wireless sensor networks are usually deployed in the harsh environment to monitor the designated area, sensor nodes may fail simultaneously and the function of the network will be affected even be destroyed. Thus, it is important and urgent for the network to restore from the failure and continue monitoring. Since the problem of single node failure contains many cases, the problems of multiple node failure must be much more complicated. The failed nodes may be adjacent to each other or be the 2-hop neighbors of each other, these situations need to be broken down into simple cases in order to handle them. To predigest the recovery process and solve this problem preferably, the designated area, which the sensor nodes are deployed in, is divided into grids [21].

**Figure 4:**
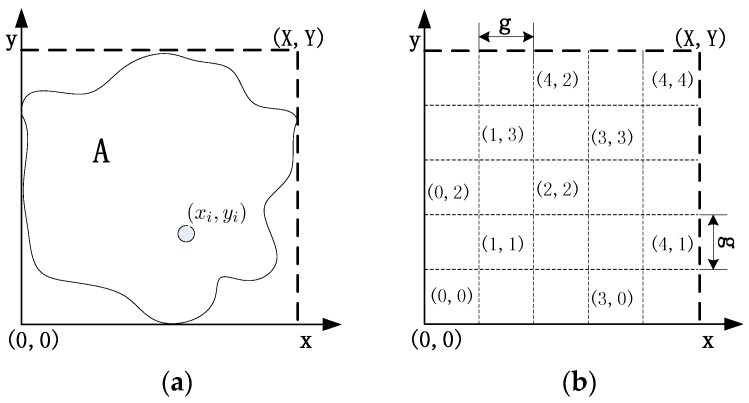
(**a**) Area sketch; and (**b**) network gridding.

As shown in Figure 4a,b, A denotes the deployed area and $\left(x_{i},y_{i}\right)$ is the coordinates of arbitrary node i. The grid that a node belongs to can be indicated as:

(7) $$G_{x}^{i}=\lfloor \frac{x_{i}}{g}\rfloor ,G_{y}^{i}=\lfloor \frac{y_{i}}{g}\rfloor ,$$

The grid size is decided by variable g; and $G_{x}^{i}$ and $G_{y}^{i}$ denote the row-coordinate and the column-coordinate of node i, respectively. After deployment, the nodes will figure out which grid they belong to using the information of the designated area. By means of gridding, every node will be ascribed to the specified grid. The number of grids will change with variable g and the multiple node failure problems will be solved in each grid severally.

#### 4.3.1. Problem Description

As mentioned above, single node failure may partition WSN into different blocks, let alone the multiple node failures. Consequently, the algorithm CCRA is proposed to solve this problem based on the algorithm CSFR-M and takes several remedial measures when CC-link is hard to construct. The main purpose of CCRA is to localize the connectivity restoration in each grid to simplify the process and restore the network connectivity with minimum number of involved nodes, called the displacement distance. Different to CSFR and CSFR-M, all of the nodes know their grid coordinates $\left(G_{x}^{i},G_{y}^{i}\right)$ and they will also maintain their 2-hop neighbors’ grid coordinates in the information table after the self-organization. Meanwhile, the inner/inter grid property of each 2-hop neighbor will be attached in the information table.

**Figure 5:**
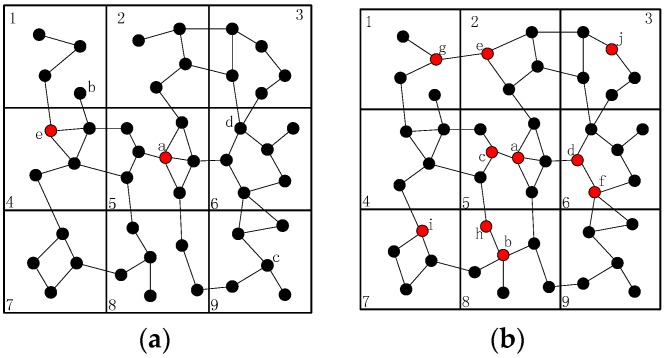
(**a**) Inner-grid node failures; and (**b**) inter-grid node failures.

**Definition** **7.** *(Inner-grid node) Node* i *is an inner-grid node if and only if all of its 1-hop neighbors are in the same grid, i.e.,* $\{\begin{array}{c} G_{x}^{i}=G_{x}^{j}\;\left(d_{i,j}\leq R_{c}\right)\\ G_{y}^{i}=G_{y}^{j}\;\left(d_{i,j}\leq R_{c}\right) \end{array}$*, such as node* a *and* c *in Figure 5a*.

**Definition** **8.** *(Inter-grid node) Node* i *is an inter-grid node if and only if its 1-hop neighbors are in different grids, i.e.,* $\exists \;G_{x}^{i}\neq G_{x}^{j}\;\left(d_{i,j}\leq R_{c}\right)\;or\;G_{y}^{i}\neq G_{y}^{j}\;\left(d_{i,j}\leq R_{c}\right)$*, such as node* b*,* d *and* e *in Figure 5a*.

As shown in Figure 5a, the loss of inner-grid node (such as node a) may cause the network to be partitioned. Upon gridding, the influence is only considered in the grid with the concept of CCRA in this paper. In addition, the inter-grid node failures, such as node e, may affect the network connectivity significantly. The failure of node e will break the connecting path between grid 1 and 4, thus grid 1 will be isolated grid and the network connectivity will be destroyed. Different from the single node failure problem, multiple nodes may fail in the neighboring area, i.e., the same grid as shown in Figure 5b. Considering all the analysis above, this problem may still be classified into two fundamental categories: the failures of inner-grid node and the failures of inter-grid node.

***Case 1:*** the failures of inner-grid node, as shown in Figure 5b. If node a and b fail simultaneously, the neighbors of them will initial the algorithm similar as CSFR-M to recover the failures respectively.

***Case 2:*** the failures of inter-grid node, as shown in Figure 5b. If node g, h and i fail simultaneously, the repeated utilization of CSFR-M can also repair the connectivity.

**Figure 6:**
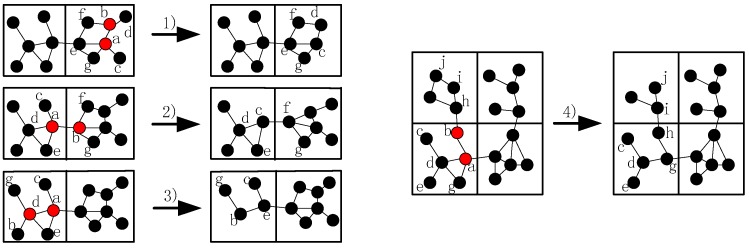
Different cases of multi-node failure.

The multiple node failure problems are the combination of these two cases, the basic principle is to ensure the connectivity inside each grid prior to the inter-grid connectivity, i.e., restore the failures of inner-grid node first in the case that the inner-grid nodes and inter-grid nodes fail simultaneously in the same grid. There are three main categories: (1) multiple nodes fail in different grids and they cannot communicate with each other directly; (2) multiple nodes fail in the same grid; and (3) multiple nodes fail in different grids but they have connecting paths. The first category is the simple combination of the two cases previously and can be handled using CSFR-M repeatedly, but the remaining are much more complicated (Figure 6):

(1) When more than one inner-grid nodes fail in the same grid, such as node a and b, as mentioned above, the restoration should be localized in the grid as far as possible. Then, the neighbors of them (denote as $N_{f}$ hereafter) will check whether they could build the CC-link first, if not, the nodes in $N_{f}$ will initial the recovery process based on the node mobility. The fundamental principle is that once the neighbor node has been selected to replace one failed node, such as node a, it can only move to replace node a and another node will be chosen as the candidate of node b.
(2) When different inter-grid nodes fail in the neighboring grid, such as node a and b, if there are no neighboring orphan nodes and the CC-link is hard to build after the failure, the neighbor node of the failed node in the same grid that has the shortest distance to the failed node will move to repair the failure. The node with least number of inter-grid neighbors will be selected if the previous parameter is the same. Finally, the smallest node ID is preferred.
(3) When inner-grid and inter-grid nodes fail in the same grid, such as node a and d, as mentioned above, the inner-grid node restoration goes first, so node d will be replaced by node b primarily in this case, then node e will move to the location of node a.
(4) When more than one inter-grid nodes fail in the same grid, such as node a and b, since each node maintains a 2-hop neighbors’ table, node h has the information of the failed nodes in this case and it can find out that one of the only two neighbors of node b (i.e., node a) has also failed, so it will estimate that there are no enough nodes to replace node b and decides to move to recover the connectivity, the topology after the restoration is shown in Figure 6.

#### 4.3.2. Algorithm Details

Most cases of multiple node failure have been discussed in the foregoing analysis, but there might still be some special cases. However, CCRA is an adaptive algorithm that it can adapt to all of these situations. When failures are detected by neighbors of the failed nodes, the recovery may be initialed simultaneously. The nodes in $N_{f}$ will decide whether there are orphan nodes after the failure firstly, if so the nearest orphan will move to restore the connectivity; if not, the nodes in $N_{f}$ will try to establish the CC-link according to Equation (3); otherwise, they will make decisions whether to move or not for restoring the connectivity. The fundamental principle is to move the nodes that have the least influence to the network topology, i.e., the node connectivity materiality is smallest and the node is nearest to the failed node. Moreover, the nodes that have minimum number of neighbor nodes are preferred. Restoration is localized in the grid as far as possible, unless too much nodes fail in the same grid and it is impossible for the remainder to reestablish the connectivity. In that case, the recovery will be operated similar to that in Case 4 previously. Since the algorithm is based on the gridding, the probability of failed nodes being located in the same grid will decrease significantly as will be demonstrated in the simulation. Finally, the pseudo-code is presented as Algorithm 4:
**Algorithm 4** CCRA**1**:   $\psi_{f}$: the set of failed nodes which need to be restored**2**:   **if** node $i\in \psi_{f}$ has no failed neighbor(s) in the same grid **then****3**:    **if** node $i\in \psi_{f}$ has no failed neighbor(s) in the neighbor grid **then****4**:     recovery process goes to case 2)**5**:    **else****6**:     restore the connectivity similar to CSFR-M**7**:    **end if****8**:   **else****9**:    **if** the connected failed nodes are all inner-grid nodes **then****10**:     recovery process goes to case 1)**11**:   **else****12**:     **if** the connected failed nodes are all inter-grid nodes **then****13**:      recovery process goes to case 4)**14**:     **else****15**:      recovery process goes to case 3)**16**:     **end if****17**:    **end if****18**: **end if**

## 5. Algorithm Analysis

The CSFR-M and CCRA algorithms are reactive processes to handle the problem of node failure(s) via cooperative communication primarily and motion ability as the assistant method when CC-link is unable to be established. The energy effectiveness of cooperative communication has been investigated by Gokturk et al. [28]. It is apparent that the node movements will consume much more energy than the communication between the nodes, thus the communicating energy consumption because the recovery process may be ignored compared to that of node movements. Since this paper is concerned on connectivity restoration, the communication between the sensor nodes is assumed with no delays and losses. Meanwhile, the sensing area and overall deployment area shrinkage are not concerned in this paper since CSFR-M and CCRA are focused on the connectivity restoration with minimum number of involved nodes.

**Lemma** **1.** *If the deployed area of WSN has been divided into grids, the failure of inner-grid node only affects the connectivity of the grid which it belongs to*.

**Proof.**  Upon gridding, the network connectivity can be expressed as the connection of all of the grids. As we can see from the definition of inner-grid node, all of the neighbors of the inner-grid node are in the same grid, i.e., the inner-grid nodes do not communicate with the nodes in the different grids directly. Since the restoration is localized in each grid, the loss of inner-grid node can be replaced by the neighbors in the same grid. The subsequent node movements, which may include the motion of inter-grid nodes, can be treated as the restoration of inter-grid nodes. That is, the influence of inner-grid node failure only locates in the grid.

**Lemma** **2.** *The recovery will be simplified significantly after gridding in CCRA*.

**Figure 7:**
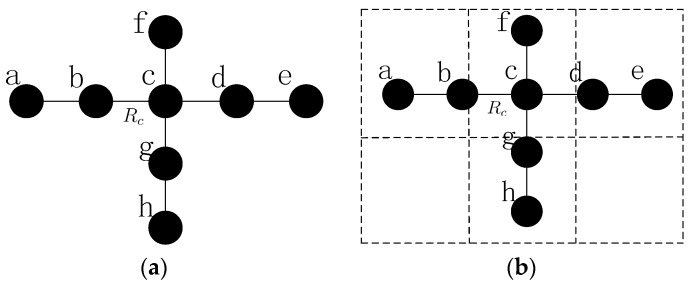
(**a**) Special case; and (**b**) special case after gridding.

**Proof.**  As shown in Figure 7a, when all the nodes are deployed in a straight line and the distances between each node are $R_{c}$. Nodes b,c and d are adjacent failed nodes, node a and e can only detect the failures of node b,c and c,d, respectively, but node f and g can detect the failures of all three nodes. The recovery process will be complicated. However, in CCRA, the failed nodes may not be in the same grid with a suitable grid size g and the restoration can be done in each grid simply, as shown in Figure 7b.

**Theorem** **1.** *The failed node f needs to be restored in CSFR-M and CCRA if $\exists \;a,b\;\in N_{f}\;and\;a\notin \;N_{b},\;s.t.\;N_{a}\cap N_{b}=f$, where $N_{f},N_{a}\;and\;N_{b}$ indicate the set of neighbor nodes of node a,b,f, respectively*.

**Figure 8:**
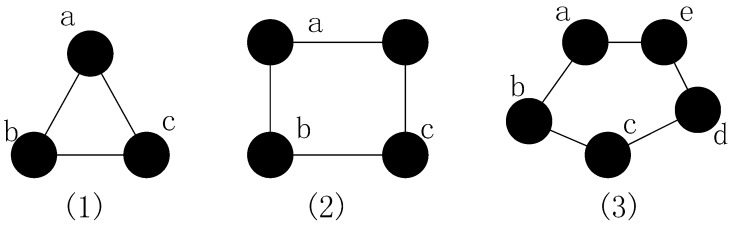
Examples.

**Proof.**  As mentioned before, CSFR-M and CCRA are not only focused on the restoration of cut-vertex, and obviously it is not necessary for some nodes to be recovered such as leaf nodes, so it is important to decide which node needs restoration. In Section 3, we bring out the node connectivity materiality $M_{f}$, which is based on the shortest path hops between the neighbors of node f before and after the failure. Apparently, the smallest number of hops of two arbitrary non-adjacent neighbors of node f is 2, and the value will grow after the failure of node f since it is the only common neighbor node. As shown in Figure 8: (1) when node a fails, the shortest path hops between node b and c is still 1 as they are adjacent; (2) when node a fails, the number of shortest path hops between node b and d maintains at 2 because they have another common neighbor, node c; and (3) the shortest path hops between b and e will increase if node a fails, so the failure of node a needs to be recovered which proves the theorem.

**Theorem** **2.** *The message complexity of the CSFR-M and CCRA are O(N), and the computation complexity of CSFR-M and CCRA are $O\left(N^{2}\right)$, where N is the number of deployed mobile sensor nodes in the WSN*.

**Proof.**  The calculation of node connectivity materiality depends on information regarding the whole network gained by the sink node during the self-organization and the message complexity is $O\left(N^{2}\right)$. The competition of source and destination nodes is among the neighbors of failed node only, the computation complexity of each node in $N_{f}$ is $O\left(n_{f}{}^{2}\right)$, where $n_{f}$ is the number of nodes in $N_{f}$ and $n_{f}=N-1$ in the worst case. Then, the determined source and destination node will check whether the CC-link can be established or not. This does not cost any exchanged messages since the decision is made by the source and destination nodes only. If CC-link can be established bilaterally, the source and destination nodes will send one informing message along with the data packet to the nodes in H(s) and H(d), where H(s) and H(d) are the helper nodes of source and destination nodes. Then, the source and helper nodes will transmit the same data packet to destination node simultaneously, and vice versa. Thus, totally, (N−3) messages will be needed in the worst case when the CC-link can be established. If CC-link cannot be built, the source and destination nodes will send one message to the nodes in $N_{f}$ announcing the failure of CC-link establishment and the selected candidate will move to replace node f. Totally, N−3 informing messages will be transmitted in the worst case. The candidate will broadcast one message to its children about its movement and N−3 nodes will move in the worst case. Thus, a total of 2×(N−3) messages would be needed when the CC-link cannot be built. Therefore, the message complexity of CSFR-M is O(N). The computation complexity of CSFR-M is $O\left(N^{2}\right)$. The analysis of message complexity and computation complexity of CCRA is similar, the message complexity is also O(N) and the computation complexity will be $O\left(N^{2}\right)$.

## 6. Simulation Results

### 6.1. Single Node Failure Restoration

In this section, we will validate the effectiveness of the CSFR algorithm through simulation and compare the proposed single node failure restoration algorithm CSFR-M with the previous algorithms DARA, PADRA and RIM. All of the experimental results are achieved on Matlab2009b with a 3.3 GHz CPU and 4 GB RAM computer. In the simulations, numerous mobile nodes are deployed randomly in an area of 500 m × 500 m with uniform communication range $R_{c}$ (i.e., all nodes are deployed with the same initial power). The following metrics are presented to evaluate the performance of CSFR and CSFR-M:

- ***Unsuccessful Repair Ratio***: The ratio of unsuccessful repair times and the times that the CSFR works. As mentioned before, the cooperative communication is established based on Equation (6). In some cases, the source node or destination node may have no enough neighbors to build the CC-link, so the restoration maybe unsuccessful.
- ***Cooperative Communication Power Ratio (PR)***: reports the ratio of the average assigned cooperative power and the initial power, where the average assigned cooperative power is the mean value of assigned cooperative power that required for the source node, destination node and their respective helper nodes to build the CC-link according to Equation (6). It is expressed as percentages in the Figures hereafter.
- ***Average Travel Distance (TD)***: The average travel distance experienced by every node that gets involved in the recovery process. The unit of measure is meter hereafter.
- ***Number of Relocated Nodes (RN)***: The average number of nodes that move during the restoration process. This metric reveals the scope of recovery process within the network and it works on CSFR-M and CCRA during the simulation.
- ***Number of Sent Messages (SN)***: The total number of messages that has been sent among the nodes during the restoration.

Meanwhile, some parameters are utilized to vary the WSN topology characteristics in different simulations and discuss the implications on the performance of CSFR and CSFR-M. They are shown as follows:

- ***Number of Deployed Nodes (DN)***: This parameter influences the node density so that the network connectivity will be affected. Since large Number of Deployed Nodes increases the node density, the number of neighbors of each node grows and it is more beneficial for establishing the CC-link.
- ***Communication Range ($R_{c}$)***: As assumed before, all deployed nodes have the same communication range $R_{c}$ and the value of $R_{c}$ is directly proportional to the initial power $P_{0}$ of each node. Small $R_{c}$ will create a sparse network topology while the large $R_{c}$ increases the connectivity of the holistic network. The CC-link can be built or not with different values of $R_{c}$ and the number of nodes which get involved in the cascaded movement during the restoration process will also be affected.

In the simulations, we have simulated different network topologies (sparse and dense) with some combinations of values of $R_{c}$ and DN. For CSFR algorithm, the value of $R_{c}$ is chosen as 140, 120, 100, 80 and 60 m, the DN is chosen from 10–100. For CSFR-M, DARA, PADRA and RIM, the value of $R_{c}$ is chosen from 10–100 m and the DN is also from 10–100. Without loss of generality, the path loss exponent ∂ in Equation (6) is set as 2. All topologies are run after detecting a failed node randomly and the result of the individual experiment is averaged over 30 tests. All results are subjected to 95% confidence interval analysis and stay within 5% of the sample mean.

**Figure 9:**
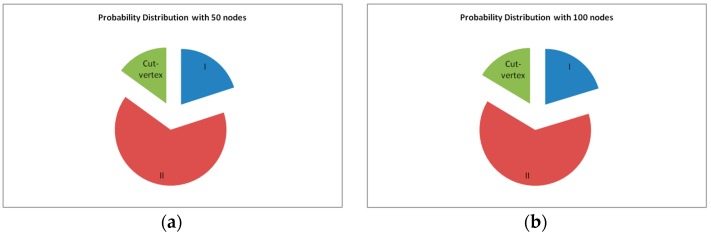
(**a**) Probability distribution with 50 nodes; and (**b**) probability distribution with 100 nodes.

Upon deploying, the influence of each node on the network connectivity is different. Since this paper focuses on the restoration of any kind of node failure(s), it is important to make the components of the network clear. Figure 9a,b show the probability distribution of categories that the nodes may belong to after deployment (the communication range $R_{c}$ is set as 100 m). The nodes with node connectivity materiality $M_{f}=1$ are classified into Category Ι; the nodes with node connectivity materiality $1<M_{f}<\infty$ are Category ΙΙ; and the nodes with node connectivity materiality $M_{f}=\infty$ are cut-vertices. As can be seen in the Figure 9a,b, it is necessary to handle the node failures in Category ΙΙ as there are many of them in the network.

#### 6.1.1. Unsuccessful Repair Ratio

**Figure 10:**
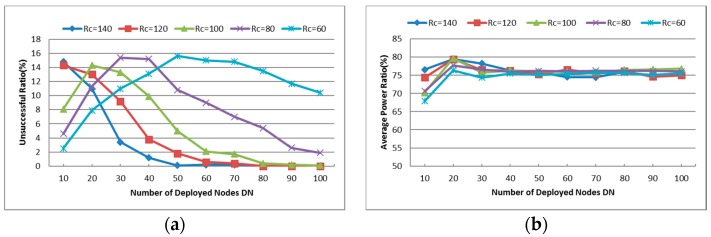
(**a**) UR with different $R_{c}$ and DN; and (**b**) PR with different $R_{c}$ and DN.

As mentioned above, this metric is only for CSFR. Figure 10a reports the Unsuccessful Repair Ratio (UR) during the restoration process under different $R_{c}$ and DN. As can be seen in Figure 10a, the UR of each value of $R_{c}$ approaches zero with the increase of DN. When $R_{c}$ is large, the UR decreases quickly as DN increases. While DN is small, the CSFR is more prone to fail when the value of $R_{c}$ is large because the network topology with smaller $R_{c}$ is sparser and it may not need any recuperation. Although there are some disadvantages in CSFR, we can see in Figure 10a that the CSFR is more effective in dense network topology.

#### 6.1.2. Cooperative Communication Power Ratio (PR)

Figure 10b captures the Cooperative Communication Power Ratio (PR) of each simulation with different values of $R_{c}$ and DN. Again, the results are the mean value over multiple simulations as mentioned above. As shown in Figure 10b, the PR of most simulations is around 75% even with different $R_{c}$ and DN. In sparse network topology (i.e., the DN is small), the number of neighbors of each node varies greatly and the PR increases as the $R_{c}$ increases. When the number of deployed nodes increases, the neighbors of each node increase. According to Equation (3), the required power for CC-link mainly depends on the distance between the source and destination nodes. When the neighbors are enough for establishing the CC-link, the required power ratios will hold at some certain values (around 75% as shown in Figure 10b). As the CSFR-M algorithm is expanding from CSFR to avoid its disadvantage, the PR metric of CSFR-M is similar to that of CSFR.

#### 6.1.3. Average Travel Distance (TD)

**Figure 11:**
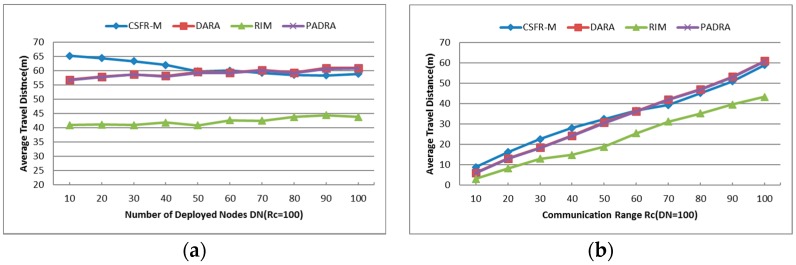
(**a**) TD with different DN; and (**b**) TD with different $R_{c}$.

Since the node movements consume more energy than communications, the minimum number of relocated nodes (RN) and average travel distance (TD) will indicate the minimum energy expense of the algorithms. AS the CSFR-M is expanding from CSFR and combining it with node mobility to improve the efficiency, the Average Travel Distance of each node that is involved in the recovery process is an important metric that assesses the performance of the algorithm. As shown in Figure 11a,b, the average travel distance of CSFR-M is compared to three previous algorithms DARA, RIM and PADRA with different values of $R_{c}$ and DN. As explained in Section 2, DARA and PADRA focus on the recovery of single cut-vertex failure, while RIM can handle any single node failure, similar to CSFR-M.

Figure 11a indicates the impact of varying DN values for the network, while all the nodes are equipped with the uniform communication range $R_{c}=100$. Small DN makes the CSFR-M a little poor than others in the performance of average travel distance, but the maximum probability of restoring the connectivity with node movement when $R_{c}=100$ is around 14% as shown in Figure 10a. With the increase of DN, the performance of CSFR-M is as good as DARA and PADRA. Considering comprehensively the performance of CSFR-M in terms of average travel distance is better than DARA and PADRA algorithms. The average travel distance of RIM is the smallest since the biggest movement distance of RIM is limited to $R_{c}/2$. Apparently, the average travel distance will increase as $R_{c}$ becomes larger, as we can see in Figure 11b. In a word, CSFR-M is better than DARA and PADRA comprehensively in the performance of average travel distance of each node.

#### 6.1.4. Number of Relocated Nodes (RN)

**Figure 12:**
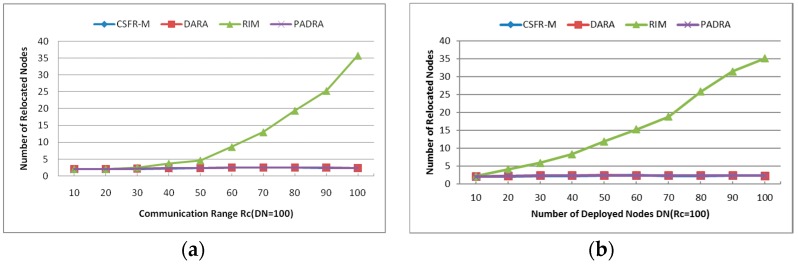
(**a**) RN with different $R_{c}$; and (**b**) RN with different DN.

The average number of relocated nodes during the restoration with different $R_{c}$ and DN is shown in Figure 12a,b. As mentioned earlier, the plotted results are the average over multiple independent simulations. The two figures indicate that CSFR-M, DARA and PADRA need almost the same nodes in the recovery, and the number of reposition nodes is fewer than RIM since RIM requires all neighbors of the failed node to move. Obviously, the number of relocated nodes in RIM will increase when the values of $R_{c}$ and DN grow because the number of neighbors of each node increases. However, the number of relocated nodes almost remains unchanged with different $R_{c}$ and DN. This will contribute to the candidate selection measure. Since the node with the minimum number of 1-hop neighbors is preferred, the number of relocated nodes will always be minimum with different $R_{c}$ and DN.

Again, the parameter of RN is counted as long as the node movement is used to restore the failure. However, CSFR-M does not always require the nodes to move and replace the failed node, as shown in Figure 10a. As can be seen in Figure 12a,b, CSFR-M performs the same as DARA and PADRA when the nodes movement recovery is needed. From the previous analysis, the DARA and PADRA algorithms can only handle single cut-vertex failure via node movement. However, as shown in Figure 10a, the unsuccessful ratio of CSFR may be ignored in dense network, i.e., CSFR-M could restore the network connectivity when arbitrary single node fails via cooperative communication rather than node movement in dense network.

#### 6.1.5. Number of Sent Messages (SN)

**Figure 13:**
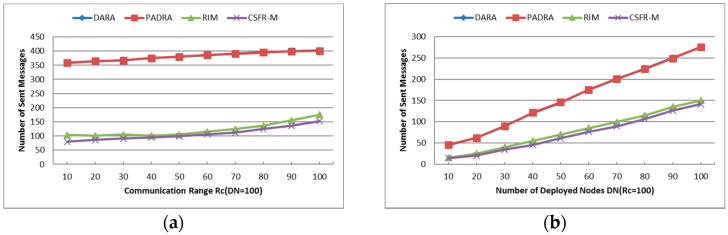
(**a**) SN with different $R_{c}$; and (**b**) SN with different DN.

As shown in Figure 13a,b, the curve of DARA and PADRA overlap with each other as they send almost the same number of messages during the restoration. The number of sent messages of CSFR-M during the restoration is a little bit less than that of RIM, but CSFR-M introduces significantly less messaging overhead in comparison of DARA and PADRA. RIM is better in the performance of this parameter because it needs 1-hop neighbor information, rather than the 2-hop neighbor information required in DARA and PADRA. Since CSFR-M only sends messages to inform the establishment of CC-link or the necessary of node movements, the messages are transmitted among a few involved nodes and the number should be small. Both figures also indicate that when the network becomes more connected, i.e., larger $R_{c}$ or DN, the message traffic grows. This can be attributed to the increased number of neighbors that must be notified before CC-link is built or relocation takes place. It is worth noting that DARA and PADRA use the same number of messages and their curves totally overlap. Considering the previous simulation results comprehensively, the CSFR-M is still an efficient and favorable approach.

### 6.2. Multiple Nodes Failure Recovery

To deal with the problems of multiple node failure, we present the collaborative connectivity restoration algorithm, CCRA, which is an extension of CSFR-M. The CCRA algorithm restores the network connectivity after multiple node failures and simplifies the recovery through network gridding. Since excessive small DN makes the network too sparse, 50–140 nodes will be deployed in an area of 500 m × 500 m with various values of $R_{c}$ from 50–140 m in the simulations. The metrics presented to evaluate the performance of CCRA are the same as CSFR-M, i.e., RN, TD and PR.

**Figure 14:**
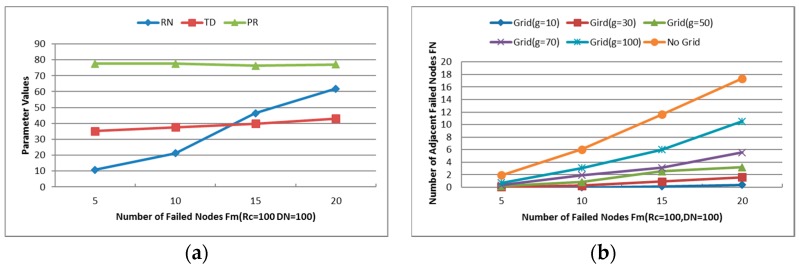
(**a**) Parameter values with different Fm; and (**b**) FN with different Fm.

Different from CSFR, CSFR-M, DARA, PADRA and RIM, CCRA deals with multiple node failure problems, and the performance of CCRA will be affected by different numbers of failed nodes (denotes as Fm). Thus, there is one more parameter to be considered as follows:

- ***Maximum Number of Failed Nodes (Fm)***: Indicates the maximum number of failed nodes in each experiment. As shown in Figure 14a, the average power ratio maintains around 75% and the average travel distance holds about 40%, but the number of relocated nodes increases significantly with the increase of Fm. When Fm is set as 20, approximately 60% of the residual healthy nodes moved in the simulations. The network topology is nearly rebuilt when so many nodes change their positions, so the next simulations will be tested with Fm = 5 and 10.

Moreover, one of the significant improvements of CCRA is gridding the network into small grids and localizing the restoration process. As described above, many neighbors of the failed nodes will move when two or more failed nodes are adjacent to each other, thus the number of adjacent failed nodes (denotes as FN) has a great influence to the performance of CCRA. Figure 14b shows the decision of the adjacent failed nodes with gridding or not when $R_{c}$ and DN are set at 100. Obviously, the number of adjacent failed nodes without gridding is larger than that with gridding in the same case, where g is the size of the grid.

**Figure 15:**
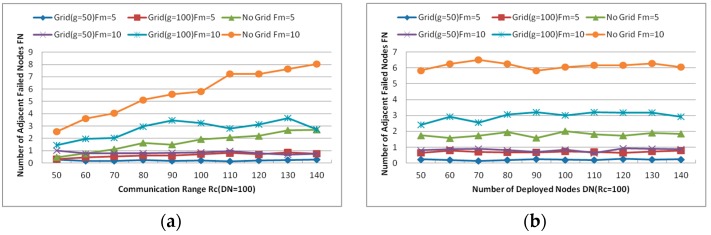
(**a**) FN with different $R_{c}$ and Fm; and (**b**) FN with different DN and Fm.

Figure 15a indicates that the number of adjacent failed nodes increases significantly without gridding in both cases (i.e., Fm = 5 and 10) when the communication range of each node varies from 30 to 140 m. However, the values of FN with gridding in both cases maintain around some fixed values. Similarly, all values of FN maintain around some fixed values in both cases, as can be seen in Figure 15b. Deliberating the results of these simulations, the values of FN are found to have smaller fluctuations with the smaller grid size g when other parameters are the same. Thus, we can image how small the values of FN could be when the grid size g is set under 50. However, as we can see from the simulation results, the value of FN is less than or equal to 1 when the grid size g=50 with different $R_{c}$ and DN. Moreover, when the grid size is excessively small, too many empty grids that do not contain any nodes will cause unnecessary troubles during the recovery process. Certainly, the computation due to gridding will be more complex when the grid size is excessively small. Hence, the grid size g will be set as 50 m in the following simulations after considering the previous analysis comprehensively and cautiously.

#### 6.2.1. Number of Relocated Nodes (RN)

**Figure 16:**
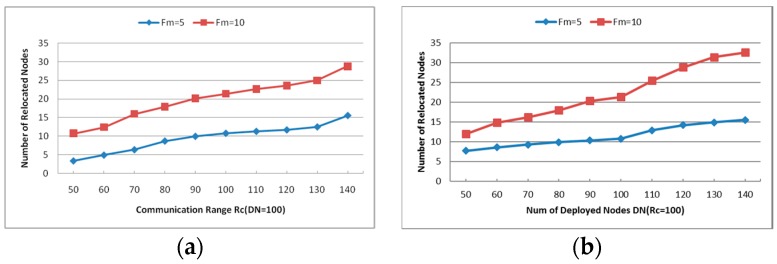
(**a**) RN with different $R_{c}$ and Fm; and (**b**) RN with different DN and Fm.

As mentioned before, the number of relocated nodes RN is the primary concern of the node failure restoration with node mobility. While the network is deployed with a fixed number of nodes (DN = 100), the number of relocated nodes increases about 10% when Fm is 5, but that of Fm = 10 increases much more obviously. However, when the communication range $R_{c}$ is set as 100, RN grows fleetly with various DN in both scenarios. Figure 16a,b indicates that when the number of failed nodes increases, the number of relocated nodes grows greatly in dense networks. Again, since the recovery process may move many residual healthy nodes to replace the failed nodes and almost rebuild the network topology, it is unnecessary to restore the network connectivity if too many nodes fail simultaneously in the network. When Fm = 5, the probability that there are neighboring failed nodes is close to zero, so the number of relocated nodes is between 5 and 15 as shown in the Figure 16a,b.

#### 6.2.2. Average Travel Distance (TD)

**Figure 17:**
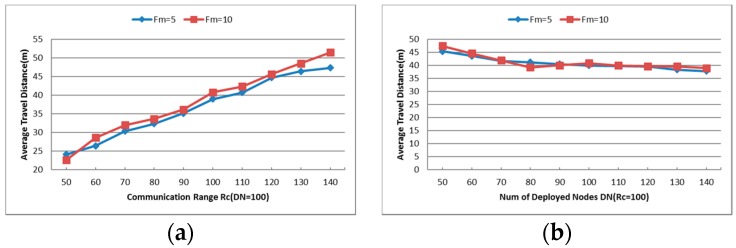
(**a**) TD with different $R_{c}$ and Fm; and (**b**) TD with different DN and Fm.

The average travel distance TD is also an important index for node failure restoration algorithm, and the results are shown in Figure 17a,b. With the increase of the communication range $R_{c}$, the average travel distance of each node rises rapidly as the number of deployed nodes is fixed. However, the situation is different when the value of $R_{c}$ is fixed. The average distance that each node travels in the recovery process remains around 40 m as the number of deployed nodes increases. As mentioned above, the primary factor that affects the recovery is the distance between the nodes, i.e., the communication range of each node influences the relations of each node significantly. Thus, different values of $R_{c}$ make the results quite different from each other.

Comparing Figure 11a,b with Figure 17a,b, it is easy to say that the average travel distance of each node in CCRA is less than that in CSFR-M, DARA and PADRA. This is because when CC-link is not useful to reestablish the connecting paths in CCRA, the suitable candidates that are nearest to the failed nodes will move to replace them as we have explained in Section 4.

#### 6.2.3. Cooperative Communication Power Ratio (PR)

**Figure 18:**
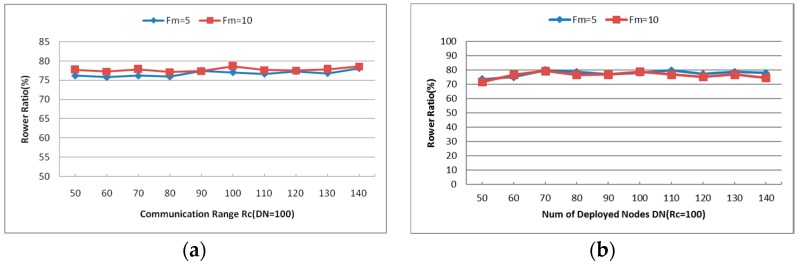
(**a**) PR with different $R_{c}$ and Fm; and (**b**) PR with different DN and Fm.

The biggest difference between CCRA and other repair algorithms is that CCRA (also CSFR-M and CSFR) use cooperative communication as the primary method to restore the network connectivity. As shown in Figure 18a,b, the cooperative communication power ratio of each involved node stays around 75%, no matter the value of $R_{c}$ changes or the number of deployed nodes is varied. Since the CCRA algorithm is restoring the network connectivity via cooperative communication primarily and moving nodes to replace the failed nodes when CC-link cannot be established, the experiment results demonstrate that the CCRA works steadily while building the cooperative paths.

Considering the above factors comprehensively, the CCRA algorithm is efficient in handling the problems of multiple node failure with the combination of cooperative communication and node mobility.

## 7. Discussion

Recently, the application of WSNs in inhospitable environment has received growing interest. In all of these applications, human intervention is hard to implement and the WSNs work unattended. Since all nodes of the WSNs are deployed in such a harsh environment, the nodes are susceptible to failure, which may influence the quality of some services and even destroy the function of the whole network. In this paper, we have researched the problem of network connectivity recovery after the failure(s) of node and presented three algorithms: CSFR, CSFR-M and CCRA. CSFR is proposed to handle the single node failure problem with cooperative communication only and it may fail due to the sparse network topology. CSFR-M is an extension of CSFR to restore the network connectivity more effectively with node motion. Moreover, CCRA is focused on the network connectivity recovery from multiple node failures. Unlike the previous schemes mentioned in the literature, CSFR-M and CCRA algorithms trigger an extensive recovery on all kinds of node through cooperative communication primarily and assistant node motion.

The performance of CSFR-M and CCRA is validated via simulation analysis. The simulation results have confirmed the effectiveness of CSFR-M in single node failure restoration and demonstrated the CCRA’s efficiency in term of multiple node failure recovery. CCRA simplifies the restoration of multiple node failures by network gridding and localizes the initial recovery in every grid with failed node(s). Additionally, the simulation results have indicated that CSFR-M and CCRA are both favorable in dense networks for which the CC-link can be established to decrease node movements.

CSFR-M can recover from arbitrary single node failure and CCRA can handle the problems of multiple node failure. The coverage loss due to the node failures has not been considered in this paper and it may be handled in our future research. In addition, the nodes are simply taken as failure nodes if any part of their functions is lost, similar to in previous literature, however, we may distinguish different failed components and make the best of the residual efficient parts for network topology reconfiguration in the future.
